# A central role for hepatic conventional dendritic cells in supporting Th2 responses during helminth infection

**DOI:** 10.1038/icb.2015.114

**Published:** 2016-01-12

**Authors:** Rachel J Lundie, Lauren M Webb, Angela K Marley, Alexander T Phythian-Adams, Peter C Cook, Lucy H Jackson-Jones, Sheila Brown, Rick M Maizels, Louis Boon, Meredith O'Keeffe, Andrew S MacDonald

**Affiliations:** 1 Institute of Immunology and Infection Research, Centre for Immunity, Infection and Evolution, University of Edinburgh, Edinburgh, UK; 2 Centre for Biomedical Research, Burnet Institute, Melbourne, VIC, Australia; 3 Manchester Collaborative Centre for Inflammation Research, University of Manchester, Manchester, UK; 4 EPIRUS Biopharmaceuticals, Utrecht, The Netherlands; 5 Department of Biochemistry and Molecular Biology, Monash University, Melbourne, VIC, Australia

## Abstract

Dendritic cells (DCs) are the key initiators of T-helper (Th) 2 immune responses against the parasitic helminth *Schistosoma mansoni*. Although the liver is one of the main sites of antigen deposition during infection with this parasite, it is not yet clear how distinct DC subtypes in this tissue respond to *S. mansoni* antigens *in vivo*, or how the liver microenvironment might influence DC function during establishment of the Th2 response. In this study, we show that hepatic DC subsets undergo distinct activation processes *in vivo* following murine infection with *S. mansoni*. Conventional DCs (cDCs) from schistosome-infected mice upregulated expression of the costimulatory molecule CD40 and were capable of priming naive CD4^+^ T cells, whereas plasmacytoid DCs (pDCs) upregulated expression of MHC class II, CD86 and CD40 but were unable to support the expansion of either naive or effector/memory CD4^+^ T cells. Importantly, *in vivo* depletion of pDCs revealed that this subset was dispensable for either maintenance or regulation of the hepatic Th2 effector response during acute *S. mansoni* infection. Our data provides strong evidence that *S. mansoni* infection favors the establishment of an immunogenic, rather than tolerogenic, liver microenvironment that conditions cDCs to initiate and maintain Th2 immunity in the context of ongoing antigen exposure.

Dendritic cells (DCs) are a heterogeneous population of pathogen-sensing antigen presenting cells (APCs) that have a central role in the initiation of immune responses and the polarization of CD4^+^ T cells.^1, 2^ Current understanding of the process of DC activation and function is heavily biased toward studies using model antigens (Ags) or components of pathogens such as bacteria, viruses or protozoan parasites that typically induce T-helper (Th) 1/Th17 responses, while the interaction between DCs and Th2-inducing organisms remains less well defined.^3, 4, 5^

Helminth parasites are the most potent natural inducers of Th2 immune responses and murine infection with *Schistosoma mansoni* is a well-characterized experimental model for studying the development of Th2 immunity *in vivo*.^4, 6^ During *S. mansoni* infection, development of the Th2 response coincides with the onset of egg production by female parasites living in the portal vasculature.^7^ *S. mansoni* eggs are metabolically active and highly immunogenic and, while many successfully exit the host by traversing the lumen of the gastrointestinal tract, some eggs are carried by the blood flow into the liver, where they become trapped in the sinusoids and induce inflammation and granuloma formation.^4, 8^ The liver is therefore one of the main sites of Ag exposure during *S. mansoni* infection and the Th2-dominated granuloma response, which serves to protect hepatocytes from toxins released by tissue-trapped eggs, is thought to be essential for host survival.^9, 10, 11^ Development of the egg-specific Th2 response begins 4–6 weeks after infection, with the peak of the ‘acute' response occurring around week 8, before a combination of regulatory mechanisms and T-cell exhaustion combine to dampen down the response during the ‘chronic' stage from approximately week 12 onwards.^4, 7, 12, 13^ Although Th2 responses are protective during the initial stages of acute schistosomiasis, prolonged production of interleukin (IL)-4/IL-13 contributes to liver inflammation, fibrosis and immunopathology during chronic infection, and host survival is dependent on mounting a balanced T-helper response.^4, 14^

Tissue-resident DCs can be broadly divided into conventional DC (cDC) and plasmacytoid DC (pDC) populations based on differential use of transcription factors for development, expression of various cell surface markers and their responses to pathogen molecules.^1, 15^ Although cDCs are highly efficient at priming naive T-cell responses, pDCs are best known for their ability to rapidly produce large amounts of type I interferons (IFN) in response to viruses, bacteria and certain toll-like receptor (TLR) agonists.^16^ In the steady state, the liver is considered an immunosuppressive or tolerogenic microenvironment, and liver-resident DCs have been reported to express lower basal levels of MHC class II and costimulatory molecules than their splenic DC counterparts.^17^ Importantly, the liver is highly enriched with pDCs, which, in their non-activated state, appear to be immunoregulatory, functioning to suppress immune responses and mediate oral tolerance *in vivo*.^18^ Furthermore, pDCs have also been shown to have an anti-inflammatory role in Th2-mediated experimental models of airway inflammation and asthma.^19^ In this context, pDCs were recruited to the lungs of allergen-challenged mice and their selective depletion enhanced Th2 cytokine production in the draining lymph nodes and exacerbated the degree of immunopathology.^19^ Whether pDCs also function to down-modulate immune responses in a Th2 infection setting is not known.

We have previously shown that CD11c^+^ DCs are the key initiators of Th2 immune responses in the liver during acute *S. mansoni* infection.^20^ Global depletion of cDC and pDC populations during the priming stage of the Th2 response against parasite eggs (weeks 4–6 post infection) markedly impaired CD4^+^ T-cell production of IL-4, IL-13 and IL-10, but had little effect on the Th1 cytokine IFN-γ.^20^ Although this work established the fundamental importance of DCs in orchestrating Th2 development against *S. mansoni* infection *in vivo*, it did not address how hepatic cDCs and pDCs respond to egg Ags or how the liver microenvironment might influence the function of DC populations in terms of their capacity to present Ag to CD4^+^ T cells. A limited analysis of the activation phenotype of DCs over the course of *S. mansoni* infection *in vivo* showed that CD11c^+^MHC class II^+^ cells isolated from the spleen displayed only minor upregulation of expression of conventional activation markers, even at the peak of the Th2 response.^21^ However, this study did not separate DC populations into cDC and pDC subsets or include a comparison of hepatic DCs isolated directly from the liver effector site.

Here we have characterized the activation status of cDCs and pDCs isolated from the liver during acute *S. mansoni* infection, both in terms of their numbers and activation state, as well as their ability to present Ag to naive or effector/memory CD4^+^ T cells. Our results demonstrate that acute *S. mansoni* infection is associated with the recruitment of both DC populations to the liver effector site and dramatic transcriptional changes to the liver microenvironment. Importantly, hepatic cDCs displayed increased CD40 expression during *S. mansoni* infection and were capable of priming naive CD4^+^ T-cell responses *ex vivo*, suggesting that they are the major DCs responsible for Th2 induction. In contrast, although pDCs isolated from the livers of *S. mansoni*-infected mice also upregulated surface markers associated with Ag presentation they were unable to support the proliferation of either naive or effector/memory CD4^+^ T cells. Notably, depletion of pDCs during murine *S. mansoni* infection did not significantly impact hepatic Th2 responses, demonstrating that pDCs neither promote nor suppress Th2 immunity in the effector site. Together, these data extend our previous work^20^ and indicate that cDCs are likely to be the critical cell population for Th2 effector cell development, function and maintenance during *S. mansoni* infection.

## Results

### *S. mansoni* infection is associated with increased numbers of DCs in the liver effector site and dramatic changes in gene expression

**Figure 1:**
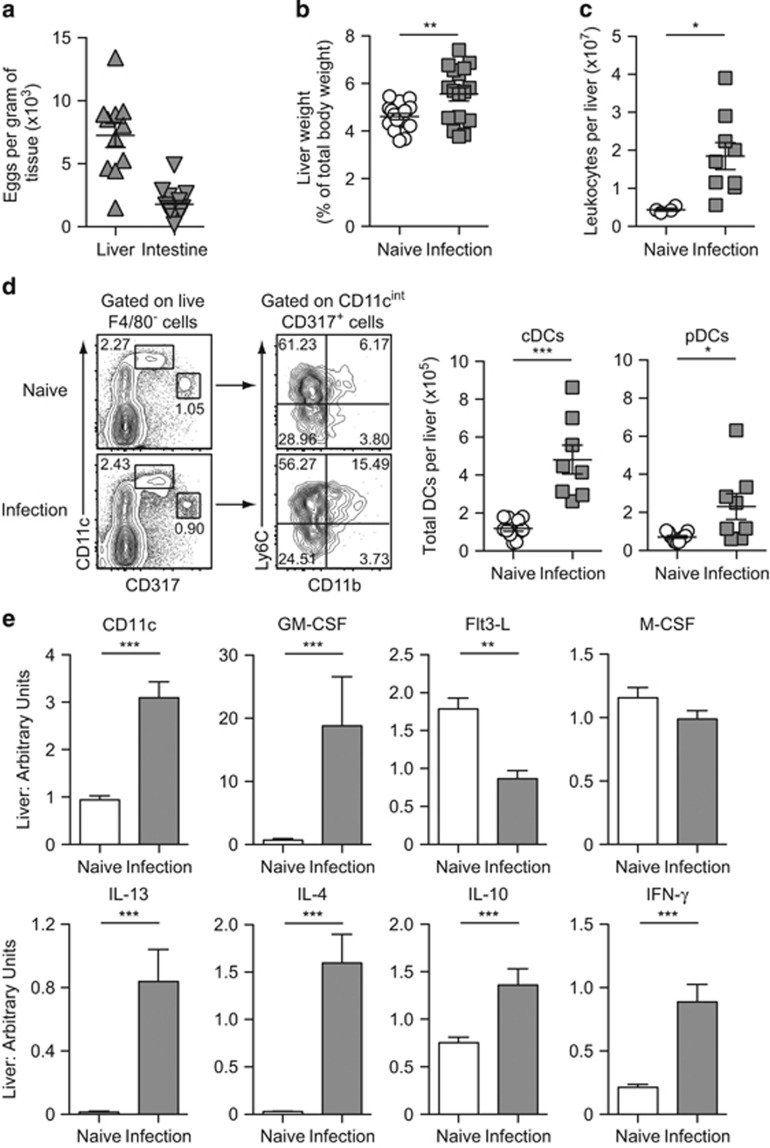
The liver is a major effector site during *S. mansoni* infection. (**a**) The total number of *S. mansoni* eggs per gram of liver or intestine tissue isolated from mice infected with *S. mansoni* for 6 weeks. (**b**) Liver weights of naive and infected mice represented as a proportion of total body weight. (**c**) The total number of leukocytes isolated from the livers of naive and infected mice. (**d**) Gating strategy to identify DC populations and quantification of the total number of cDCs and pDCs in the livers of naive and infected mice. The cDC population was defined as F4/80^−^CD11c^high^CD317^−^ cells, whereas the pDC population was defined as F4/80^−^CD11c^int^CD317^+^Ly6C^+^CD11b^−^ cells. (**e**) Quantitative RT-PCR was used to measure mRNA transcripts in whole-liver tissue from naive and infected mice. Data are expressed relative to the housekeeping gene *Ubiquitin*. Data are pooled from two experiments. Error bars indicate mean±s.e.m.

The liver is one of the principal effector sites during *S. mansoni* infection, with parasite eggs that are carried there by the blood flow becoming trapped in the sinusoids and forming the foci of immune-mediated granulomas.^4^ To investigate the impact of *S. mansoni* infection on hepatic DC populations during the acute phase of disease, livers were harvested from mice 6 weeks after infection for enumeration of DC numbers and characterization of gene expression in the liver microenvironment. This time point was selected based on our previous work, which demonstrated that CD11c^+^ DCs were critical for Th2 induction at weeks 4–6 post infection.^20^ As expected, *S. mansoni* eggs were present in both livers and intestines of infected mice, confirming the successful establishment of infection (Figure 1a). Furthermore, acute *S. mansoni* infection was associated with hepatomegaly, defined as an increase in liver weight as a proportion of overall body weight (Figure 1b), and an increase in the total number of leukocytes present in the liver (Figure 1c). To quantify the total number of DCs in the liver, the number of viable leukocytes was multiplied by the percentage of cDCs (F4/80^−^CD11c^high^CD317^−^) and pDCs (F4/80^−^CD11c^int^CD317^+^Ly6C^high^CD11b^−^) as determined by flow cytometry (Figure 1d). Importantly, although there were no significant differences between the proportion of cDCs (naive 2.31±0.34% vs infected 2.14±0.38% *P*=0.7532) and pDCs (naive 1.21±0.13% vs infected 0.75±0.20% *P*=0.0585) in the liver preparations, there was a significant increase in the total number of both populations (Figure 1d), indicating that DCs are recruited to, or differentiate within, this major effector site during acute *S. mansoni* infection.

To characterize gene expression in the liver microenvironment at this stage of the response to *S. mansoni* infection, RNA was purified from whole-liver tissue and quantitative reverse transcription PCR (RT-PCR) was performed to determine transcript levels of key DC and macrophage growth factors and cytokines that drive regulatory/Th1/Th2 immune responses.^6, 22^ Consistent with the increased number of DCs in the liver at week 6 post infection (Figure 1d), gene transcripts for both CD11c and granulocyte/macrophage colony-stimulating factor (GM-CSF) were significantly upregulated at this time point (Figure 1e), indicating that the liver microenvironment favors the differentiation and proliferation of myeloid lineage cells^23^ during establishment of the Th2 response. In contrast, acute *S. mansoni* infection was associated with a significant downregulation in transcription of FMS-related tyrosine kinase 3 ligand (Flt3-L), a cytokine that mobilizes various DC subsets *in vivo* and promotes expansion of both cDC and pDC subsets from progenitor cells^24, 25, 26^ (Figure 1e). Importantly, M-CSF gene expression was not altered during acute *S. mansoni* infection (Figure 1e), consistent with its role in promoting the differentiation and survival of macrophage populations that further the development of liver fibrosis during the later stages of disease.^27^ Acute *S. mansoni* infection also had dramatic effects on the Th2 and regulatory cytokine milieu in the liver, with elevated transcription of genes encoding IL-4, IL-13 and IL-10 detected at week 6 post infection, as expected (Figure 1e). Similarly, expression of IFN-γ was also elevated in infected livers (Figure 1e), consistent with the characteristic mixed Th1/Th2 immune response induced against the parasites at this time point.^4^

### Hepatic DCs respond to *S. mansoni* by upregulating surface markers associated with Ag presentation

**Figure 2:**
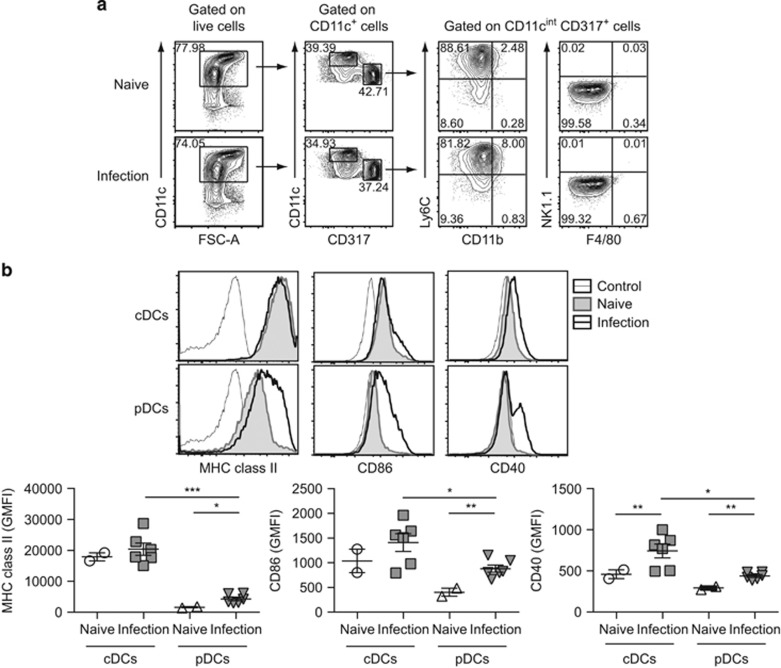
Hepatic DCs upregulate markers associated with Ag presentation and T-cell costimulation during *S. mansoni* infection. (**a**) Gating strategy to define cDCs and pDCs enriched from the livers of naive mice and mice infected for 6 weeks with *S. mansoni*. Following depletion of lineage negative cells, cDCs were defined as CD11c^high^CD317^−^ cells, whereas pDCs were defined as CD11c^int^CD317^+^Ly6C^+^CD11b^−^NK1.1^-^F4/80^−^ cells. (**b**) The geometric mean fluorescence intensity (GMFI) values for MHC class II, CD86 and CD40 on cDCs and pDCs enriched from the livers of naive or infected mice. Data are pooled from two experiments. Error bars indicate mean±s.e.m.

To examine the activation phenotype of the DC subsets recruited to the liver at week 6 post infection, CD11c^+^ DCs were enriched from liver leukocyte preparations, subdivided into cDC and pDC populations based on surface markers CD11c, CD317 (PDCA-1/BST-2), Ly6C, CD11b, NK1.1 and F4/80 (Figure 2a), and then assessed for expression of MHC class II and costimulatory molecules CD86 and CD40 by flow cytometry (Figure 2b). Although hepatic cDCs did not upregulate MHC class II or CD86 in response to infection, surface levels of these markers were significantly increased on hepatic pDCs isolated from *S. mansoni*-infected mice compared with naive mice (Figure 2b). In contrast, CD40 expression was upregulated on both cDCs and pDCs isolated from infected livers (Figure 2b), consistent with a requirement for CD40:CD154 interaction for Th2 induction *in vivo*.^21, 28, 29^ Importantly, overall marker expression remained significantly higher on cDCs, despite infection-induced activation of the pDC population (Figure 2b).

As a comparison, DCs were also purified from the spleen and their activation phenotype examined at week 6 of *S. mansoni* infection. Splenic cDCs expressed significantly higher levels of CD40 during infection, however, consistent with previous work,^21^ MHC class II and CD86 expression remained unchanged compared with naive controls (Supplementary Figure 1). It is likely that our more refined DC enrichment protocol, combined with further separation of CD11c^+^ cells into cDC and pDC subsets, accounted for our ability to detect the subtle but statistically significant changes in cDC CD40 expression at this time point during *S. mansoni* infection. MHC class II, CD86 and CD40 expression on splenic pDCs remained unchanged during *S. mansoni* infection (Supplementary Figure 1), indicating that the liver is a major site of pDC activation in this infection model. Consistent with the liver data (Figure 2b), the overall levels of expression of these three surface markers were significantly higher on splenic cDCs compared with pDCs (Supplementary Figure 1). Together, these data demonstrate that DC populations have distinct activation profiles in response to *S. mansoni* depending on their anatomical location. Furthermore, the upregulation of surface markers associated with Ag presentation and T-cell costimulation on pDCs isolated from the liver during *S. mansoni* infection suggests that this population might have an immunogenic, rather than tolerogenic, role in Th2 infection settings.

### Hepatic cDCs isolated from *S. mansoni* infection are highly efficient professional APCs

**Figure 3:**
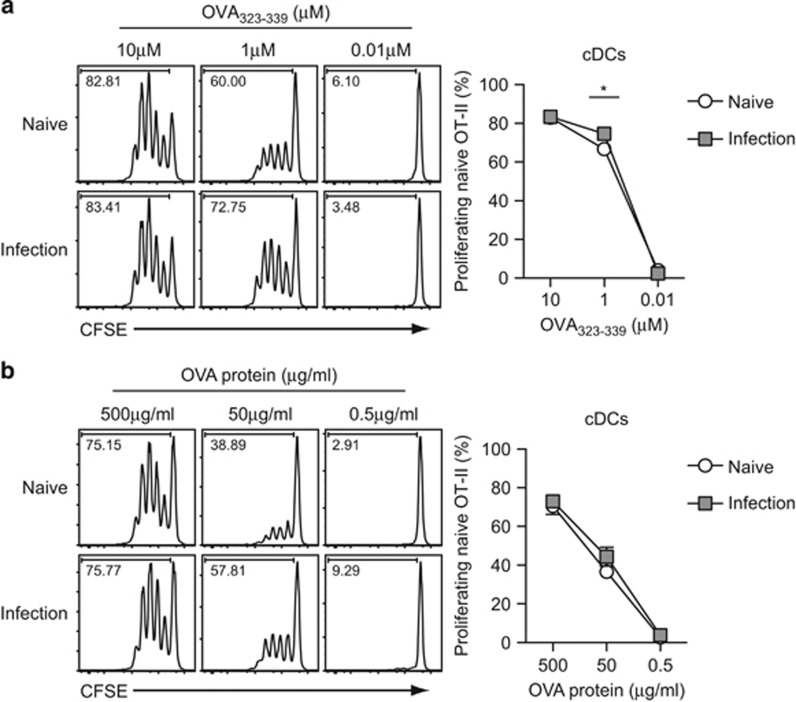
Hepatic cDCs isolated from *S. mansoni*-infected mice prime naive CD4^+^ T cells. Proliferation of naive OT-II CD4^+^ T cells in response to presentation of OVA_323–339_ peptide (**a**) or soluble OVA protein (**b**) by cDCs isolated from the livers of naïve or infected mice (6 weeks post infection). Data are pooled from three experiments. Error bars indicate mean±s.e.m.

As hepatic DCs upregulated MHC and costimulatory molecules associated with Ag presentation during *S. mansoni* infection (Figure 2), we next investigated their ability to prime naive CD4^+^ T-cell responses *ex vivo*. To do this, DCs were purified from the livers of naive or infected mice, FACS (fluorescence-activated cell sorting) purified into cDC (CD11c^+^CD317^−^F4/80^−^NK1.1^−^) and pDC (CD11c^+^CD317^+^F4/80^-^NK1.1^−^) populations (>97% purity), and assessed for their ability to present Ags to naive CD4^+^ T cells using a well-described transgenic CD4^+^ OT-II T-cell co-culture system.^30, 31, 32^ This approach was necessary due to a lack of schistosome-specific TCR transgenic mice. Hepatic cDCs from either naive or *S. mansoni*-infected mice were highly efficient at inducing naive OT-II T-cell proliferation *in vitro* in response to unrelated (third-party) peptide and protein Ags, indicating that, despite a relatively ‘muted' activation phenotype,^33, 34, 35^ the Ag uptake, processing and presenting function of these cDCs was not compromised during *S. mansoni* infection (Figure 3). In contrast, hepatic pDCs from *S. mansoni*-infected mice were unable to induce proliferation of naive OT-II T cells, even at high concentrations of ovalbumin (OVA) peptide or protein (Supplementary Figures 2a and b). This was likely due to the overall lower levels of surface expression of MHC class II and costimulatory molecules on pDCs compared with cDCs isolated from infected livers (Figure 2b), and is consistent with previous studies demonstrating that pDCs are poor initiators of naive T-cell proliferation in response to exogenous Ags.^16, 30, 36, 37, 38^

### Hepatic cDCs isolated from *S. mansoni* infection support effector/memory CD4^+^ T-cell responses

**Figure 4:**
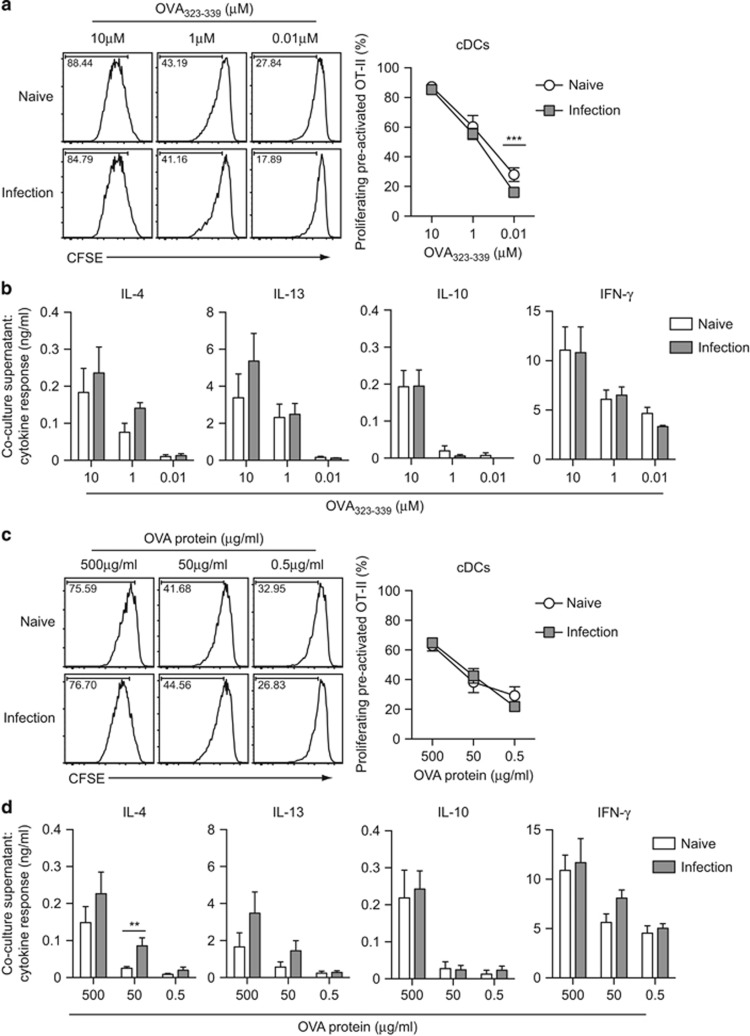
Hepatic cDCs isolated from *S. mansoni*-infected mice support effector/memory CD4^+^ T-cell proliferation and cytokine production. Proliferation and cytokine profiles of pre-activated effector/memory OT-II CD4^+^ T cells in response to presentation of OVA_323-339_ peptide (**a**, **b**) or soluble OVA protein (**c**, **d**) by cDCs isolated from the livers of naïve or infected mice (6 weeks post infection). Data are pooled from three experiments. Error bars indicate mean±s.e.m.

In addition to their unrivaled ability to activate naive T cells, DCs can also function to direct and maintain effector T-cell responses.^1, 20^ As the liver acts as a major effector site wherein effector/memory CD4^+^ T cells are continuously exposed to schistosome eggs and egg Ags,^4^ we next investigated the ability of hepatic DCs to support effector/memory-like CD4^+^ T-cell responses *ex vivo*. Purified cDCs and pDCs were sorted from naive or *S. mansoni*-infected livers and assessed for their ability to present both peptide and protein Ags to pre-activated effector/memory-like OT-II cells *in vitro.* Once again, cDCs from *S. mansoni*-infected mice were fully functional APCs, able to support the expansion of pre-activated OT-II T cells as effectively as cDCs from naive mice, in response to either peptide or protein Ags (Figures 4a and c). Furthermore, pre-activated OT-II cells produced equivalent amounts of IL-4, IL-13, IL-10 and IFN-γ after co-culture with OVA peptide or protein and cDCs isolated from the livers of either naive or infected mice (Figures 4b and d). This indicates that *S. mansoni* infection does not alter the fundamental ability of hepatic cDCs to process and present Ag to support effector/memory CD4^+^ T-cell responses. In contrast, hepatic pDCs from naive or *S. mansoni*-infected mice activated the proliferation of only a small proportion of pre-activated OT-II cells, and only at the highest concentration of peptide tested (10 μM; Supplementary Figures 2c and d). Consequently, cytokine production in pDC co-cultures was markedly reduced compared with that of cDC co-cultures (Supplementary Figures 2e and f). Interestingly, however, IL-13 production by pre-activated OT-II cells was significantly enhanced in the presence of pDCs isolated from *S. mansoni*-infected livers (Supplementary Figure 2e), suggesting that pDCs might have a supporting role in shaping some aspects of the Th2 response during infection.

### The impact of *in vivo* pDC depletion on development of Th2 immunity against *S. mansoni*

To determine whether pDCs influence any aspect of Th2 development *in vivo* during active *S. mansoni* infection, we specifically depleted this DC population using two well-established approaches. Firstly, B6 mice were treated with the pDC-depleting monoclonal antibody 120G8 every 48 h from day 28 (week 4) to day 42 (week 6) after *S. mansoni* infection. Although this treatment regime successfully depleted 60–70% of pDCs from the spleen (Supplementary Figure 3a), as previously reported,^39^ it was much less effective at depleting pDCs from the liver, where 120G8-treated mice displayed only a 15–20% reduction in hepatic pDCs compared with isotype control-treated mice (Supplementary Figure 3b). Analysis of schistosome-specific (SEA) recall responses revealed that this level of pDC depletion had no impact on the Th2 and regulatory cytokine response by cultured leukocytes from the liver (Supplementary Figure 3c; similar results were obtained for IFN-γ (data not shown)). Intracellular cytokine staining further confirmed that the ability of hepatic CD4^+^ T cells to produce IL-4, IL-13 or IL-10 in response to phorbol myristate acetate and ionomycin stimulation was not altered in 120G8-treated mice (Supplementary Figure 3d).

**Figure 5:**
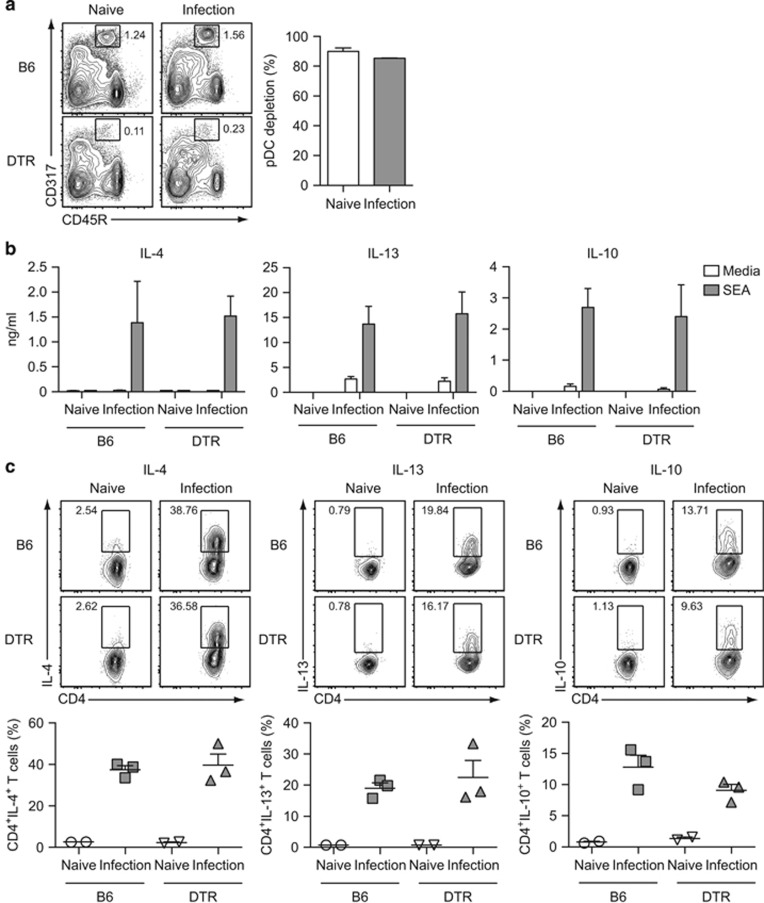
pDC depletion has no impact on Th2 responses in the liver. (**a**) DT treatment effectively depleted the CD11c^int^CD317^+^CD45R^+^ pDC population from the livers of BDCA2-DTR mice, when administered every 48 h from day 32 to day 40 post infection. (**b**) Cells isolated from the livers of naive or infected DT-treated B6 or BDCA2-DTR mice were restimulated with medium alone or SEA and supernatants were analyzed by enzyme-linked immunosorbent assay for schistosome egg-specific recall responses. (**c**) Intracellular cytokine staining was used to directly assess liver CD4^+^ T-cell cytokine production. Data represent one of three experiments. Error bars indicate mean±s.e.m.

Due to the inefficiency of the 120G8 monoclonal antibody depletion strategy in the liver effector site (Supplementary Figure 3b), we next used an alternative approach to deplete pDCs using BDCA2-DTR (blood dendritic cell Ag 2-diphtheria toxin receptor) transgenic mice, which express the DTR under the control of the human pDC gene promoter, BDCA2.^40^ Administration of DT every 48 h from day 32 to day 40 after *S. mansoni* infection significantly depleted pDCs from the livers of naive and *S. mansoni*-infected BDCA2-DTR transgenic mice, with >80% efficacy (Figure 5a). Importantly, DT treatment was highly specific for pDCs and had no measurable impact on cDCs, Ly6C^hi^ monocytes or F4/80^+^ macrophage populations (Supplementary Figure 4). Additionally, pDC depletion did not affect overall egg burdens, the total number of leukocytes, or CD4^+^CD25^+^Foxp3^+^ regulatory T cells present in the liver (data not shown). Analysis of *S. mansoni*-specific recall responses showed that the high level of pDC depletion achieved in the BDCA2-DTR transgenic mice had no significant impact on the Th2 cytokine response by cultured liver leukocytes (Figure 5b; similar data were obtained for IFN-γ (data not shown)). These results were confirmed using intracellular cytokine staining to directly assess *ex vivo* cytokine production by hepatic CD4^+^ T cells (Figure 5c and data not shown for IFN-γ). Together, these data demonstrate that pDCs do not have a major role in promoting, sustaining or regulating Th2 immunity in the liver between weeks 4 and 6 of *S. mansoni* infection.

## Discussion

*S. mansoni* parasitic helminths are complex, multicellular pathogens that induce a Th2 immune profile characterized by production of the cytokines IL-4, IL-5, IL-13 and IL-10 by CD4^+^ T cells. Although the precise molecular events and signaling pathways leading to Th2 cell differentiation *in vivo* remain poorly defined, we and others have demonstrated that the initiation of adaptive Th2 responses against helminths or allergens is dependent on and driven by CD11c^+^ DCs.^20, 41, 42, 43^ However, much of our knowledge of the interaction between Th2-inducing pathogens such as *S. mansoni* and DCs is derived from studies using bone marrow-derived DCs generated *in vitro*, which capably induce Th2 responses despite displaying a non-classical maturation phenotype following exposure to schistosome Ags.^33, 34, 35^ In the present study, we have examined the direct impact of acute *S. mansoni* infection on the two major functional classes of CD11c^+^ DCs found in the liver effector site *in vivo*. We have conducted a comprehensive assessment of the liver microenvironment at week 6 post infection, including transcript levels of known DC growth factors and Th1/Th2/regulatory cytokines, enumeration of cDC and pDC numbers, evaluation of their activation states and—most importantly—functional analysis of their ability to act as professional APCs *ex vivo* and *in vivo*. Our data suggest that, although the liver is a site of activation of both cDCs and pDCs, it is hepatic cDCs that are likely to have a key role in CD4^+^ T-cell activation and Th2 immunity during acute *S. mansoni* infection.

At ~6 weeks post *S. mansoni* infection, mature female worms living in the portal vasculature are releasing eggs, the soluble Ags of which are highly immunogenic and promote Th2 responses.^4, 7^ Eggs that become trapped in the liver sinusoids cause dramatic changes to the overall liver microenvironment, including the development of granulomas, composed of CD4^+^ T cells, macrophages, eosinophils and CD11c^+^ DCs, around the individual eggs.^4, 11^ In our study, *S. mansoni* infection was associated with hepatomegaly and an increase in the total number of leukocytes in the liver (Figures 1b and c). As a result of this enhanced immune response, overall numbers of both cDCs and pDCs were also increased in the liver effector site at 6 weeks post infection (Figure 1d), indicating expansion or recruitment of these APCs in response to *S. mansoni* egg Ags. This is likely driven by elevated expression of the growth factor GM-CSF (Figure 1e), which has a known role in recruitment, development and homeostasis of non-lymphoid tissue DCs.^41, 44, 45^

This is the first study to characterize the activation phenotype of cDC and pDC populations isolated directly *ex vivo* from the liver during *S. mansoni* infection. Our data demonstrate that hepatic DC populations display distinct activation profiles following exposure to *S. mansoni* parasites; whereas cDCs exhibited increased levels of the costimulatory molecule CD40 during establishment of the Th2 response, pDCs responded to *S. mansoni* infection by upregulating surface expression of MHC class II, CD40 and CD86 (Figure 2b). Interestingly, pDCs from the spleen displayed more limited phenotypic activation at the 6 week time point (Supplementary Figure 1a), indicating that the liver is a major site of pDC activation at this acute stage of *S. mansoni* infection. Importantly, our data demonstrating that MHC class II expression was not significantly upregulated on the surface of hepatic or splenic cDCs in response to *S. mansoni* parasites is consistent with previous work examining the activation status of splenic CD11c^+^MHC II^+^ cells over the course of *S. mansoni* infection.^21^ Although Straw *et al.*^21^ also reported no significant changes in CD40 expression at week 6 post infection, our refined DC enrichment protocol and extensive panel of cell surface markers to further subdivide the CD11c^+^ DC populations revealed a significant increase in CD40 expression on both hepatic and splenic cDCs (Figure 2b and Supplementary Figure 1a). As the focus of our study was to address the role of hepatic DCs in the early phase of the Th2 immune response to *S. mansoni* parasites, we concentrated only on the week 6 time point. However, it is highly likely that DC populations in the liver and spleen will undergo dynamic changes as the infection progresses from acute to chronic stage.

A central role for hepatic cDCs in initiating Th2 immune responses during *S. mansoni* infection was first supported by our *ex vivo* studies in which hepatic cDCs, but not pDCs, isolated from infected mice were found to be highly effective professional APCs, efficient at processing and presenting Ag to both naive and effector/memory CD4^+^ T cells (Figures 3 and 4). In addition to priming naive T cells and promoting the expansion of effector/memory T cells, cDCs from *S. mansoni*-infected livers also effectively supported Th1/Th2 cytokine production by ‘unpolarized' effector/memory T cells (Figure 4). These data indicate that *S. mansoni* does not compromise the uptake, processing or presentation of Ag by hepatic cDCs, or their ability to promote adaptive immune responses, during acute infection. As helminth infections are generally chronic, further studies are required to determine whether soluble proteins released from schistosome eggs trapped in the liver have long-term immunomodulatory effects on the ability of hepatic cDCs to initiate and maintain adaptive immune responses. This will be particularly important for understanding the potential negative impact of helminths on immune responses to vaccines and other major pathogens that coexist in schistosome endemic areas.^46^

In comparison to hepatic cDCs, pDCs from *S. mansoni*-infected livers displayed significantly lower absolute levels of expression of MHC II and costimulatory molecules (Figure 2) and, in functional terms, this resulted in poor naive T-cell stimulatory capacity *ex vivo* (Supplementary Figures 2a and b). This can been attributed to the continuous synthesis of MHC II molecules and turnover of MHC II-peptide complexes in activated pDCs, which continues long after activation, rendering this DC population inefficient in the presentation of exogenous Ags but still capable of presenting intracellular Ags in their activated state.^38^ Our results are also consistent with published studies demonstrating the poor ability of *ex vivo*-isolated pDCs to present Ags to naive CD4^+^ T cells in the context of both Th1 and allergic Th2 immune responses.^16, 30, 36, 37, 38^ Intriguingly, hepatic pDCs isolated from naive or infected mice were equally poor at supporting the expansion of effector/memory CD4^+^ T cells (Supplementary Figures 2c and d), which respond to lower doses of Ag and are less dependent on DC costimulation than naive CD4^+^ T cells.^47^ However, pDCs from *S. mansoni*-infected mice showed increased ability to support low level IL-13 production by effector/memory OT-II T cells at the highest pDC:OVA_323–339_ peptide tested (Supplementary Figure 2e). These data imply that schistosome infection may confer on hepatic pDCs a limited ability to support some key facets of Th2 functionality.

Although our *ex vivo* sorting experiments strongly suggested that hepatic pDCs from schistosome infection did not have a dominant role in Ag processing and presentation to CD4^+^ T cells, it was important to investigate the contribution of this DC population to the development of Th2 immunity during active infection *in vivo*. Schistosome-specific Th2 recall responses in the liver were neither reduced nor enhanced in pDC-depleted mice compared with controls (Figure 5). These novel data build upon our previous work showing that global depletion of both cDCs and pDCs dramatically impairs the hepatic Th2 response during murine schistosome infection,^20^ strongly suggesting that cDCs rather than pDCs are likely to be the major APCs responsible for Th2 induction during *S. mansoni* infection. Our data are in agreement with a study demonstrating that depletion of lung pDCs had no impact on the priming of naive CD4^+^ T cells in an allergic Th2 model of mouse asthma.^36^ In that setting, where pDCs failed to induce T-cell division, they functioned to downregulate the immune response by suppressing the generation of effector T cells.^36^ In contrast, in our studies we found no evidence that pDCs from *S. mansoni*-infected livers inhibited the Th2 activating ability of cDCs: firstly, in our DC:OT-II T-cell co-culture experiments, CD4^+^ T-cell proliferation was equally efficient in the presence of cDCs alone or following re-addition of pDCs (1:1 ratio; data not shown); secondly, Th2 cytokines were not elevated following pDC depletion of schistosome-infected mice (Figure 5). However, we cannot rule out the possibility that pDCs may develop tolerogenic capacity at later stages of *S. mansoni* infection.

It remains to be determined precisely which cDC subtype(s) are responsible for Th2 initiation during *S. mansoni* infection. Two recent studies have identified a specialized subset of CD11b^+^ cDCs that promote Th2 differentiation in the lung (in response to innocuous allergens) or in the skin-draining lymph nodes (in response to the parasitic helminth *Nippostrongylus brasiliensis*).^48, 49^ In these studies, Th2 induction was dependent upon DC-specific expression of the transcription factor IFN regulatory factor 4,^48, 49^ and further investigation is now required to assess whether *S. mansoni*-specific Th2 responses are also mediated by an IFN regulatory factor 4-dependent population of hepatic cDCs. Connor *et al.*^50^ have also recently demonstrated that CD11c^+^ MHC class II^+^ DCs from the skin-draining lymph nodes of *N. brasiliensis*-treated mice upregulated expression of IFR4, programmed death ligand 2 and CD301b, and acquired the ability to prime IL-4 responses *in vivo* without the cooperation of additional cell populations. This data supports the notion that DCs exposed to the appropriate parasite-conditioned environment express all of the signals required to instruct Th2 differentiation.^50^

In conclusion, this is the first comprehensive study of the activation phenotype and function of hepatic DC populations during infection with a Th2-inducing pathogen. Our data suggest that, despite a low level of phenotypic activation, cDCs are capable of stimulating naive and effector/memory CD4^+^ T cell responses and supporting hepatic Th2 immunity during acute *S. mansoni* infection. Furthermore, our results demonstrate that pDCs neither promote nor regulate hepatic CD4^+^ T cell responses at this stage of infection. We propose that the liver microenvironment conditions recruited and/or resident cDCs to support the induction and maintenance of both naive and effector Th2 responses. This would ensure that effective Th2 immunity is generated in the face of persistent and ongoing Ag exposure, which is critical for host survival against this chronic helminth infection.

## Methods

### Animals, infections and immunizations

C57BL/6 (B6) mice, and B6 background transgenic OT-II and BDCA2-DTR^40^ mice, were bred and maintained under specific-pathogen-free conditions at the University of Edinburgh, UK. Experimental mice were infected percutaneously with ∼80 *S. mansoni* cercariae from *Biomphalaria glabrata* snails. For the first pDC depletion strategy, B6 mice were injected i.p. every 48 h from day 28 to 40 with 200 μg 120G8 or IgG1 control monoclonal antibodies (courtesy of L. Boon, EPIRUS Biopharmaceuticals, Utrecht, The Netherlands). For the second pDC depletion strategy, BDCA2-DTR mice were injected i.p. every 48 h from day 32 to 40 with 8 ng g^−1^ DT (Sigma-Aldrich, St Louis, MO, USA) in phosphate-buffered saline. Endotoxin-free soluble egg Ag (SEA) was prepared in-house from *S. mansoni* eggs harvested from the livers of infected B6 mice as previously described.^33^ All experiments were approved by Project Licences granted by the Home Office (UK) and were conducted in accordance with local guidelines.

### *S. mansoni* egg counts

Livers and intestines from infected mice were digested in 4% potassium hydroxide (15 ml g^−1^ of liver tissue; 7.5 ml g^−1^ of intestine tissue) at 37 °C overnight. A total of 100 μl aliquots of the digests were evaluated on gridded Petri dishes and the eggs counted at 10x magnification. Each digest was examined in triplicate and the mean results were used to extrapolate the total number of eggs per gram of tissue.

### RNA isolation and RT-PCR

Total RNA from liver tissue was prepared using TRIzol reagent (Invitrogen, Carlsbad, CA, USA) and the RNeasy Mini Kit (Qiagen, Venlo, Limburg, The Netherlands). RNA was translated into cDNA using Superscript III Reverse Transcriptase and Oligo (dT) (Invitrogen). Quantitative RT-PCR was performed using a Light Cycler 480 II Real-Time PCR machine (Roche, Nutley, NJ, USA) and LightCycler-DNA master SYBR Green I (Roche). The relative amounts of mRNA for genes of interest were normalized to Ubiquitin. The following primers were used: Ubiquitin, 5′-TGGCTATTAATTATTCGGTCTGCAT-3′, 5′-GCAAGTGGCTAGAGTGCAGAGTAA-3′ CD11c, 5′-ATGGAGCCTCAAGACAGGAC-3′, 5′-GGATCTGGGATGCTGAAATC-3′ GM-CSF, 5′-GCATGTAGAGGCCATCAAAGA-3′, 5′-CGGGTCTGCACACATGTTA-3′ Flt3-L, 5′-CCTAGGATGCGAGCCTTGT-3′, 5′-TGTTTTGGTTCCCAACTCG-3′ M-CSF, 5′-CAACAGCTTTGCTAAGTGCTCTA-3′, 5′-CACTGCTAGGGGTGGCTTTA-3′ IL-10, 5′-CAGAGCCACATGCTCCTAGA-3′, 5′-TGTCCAGCTGGTCCTTTGTT-3′ IL-4, 5′-GAGAGATCATCGGCATTTTGA-3′, 5′-TCTGTGGTGTTCTTCGTTGC-3′ IL-13, 5′-CCTCTGACCCTTAAGGAGCTTAT-3′, 5′-CCTCTGACCCTTAAGGAGCTTAT-3′ IFN-γ, 5′-GGAGGAACTGGCAAAAGGAT-3′, 5′- TTCAAGACTTCAAAGAGTCTGAGG-3′.

### *Ex vivo* DC enrichment and flow cytometric sorting

Spleen and liver tissues were harvested from citrate saline buffer-perfused mice and digested at 37 °C (with tilting and shaking) for 20 min (spleen) or 45 min (liver) with 0.4 U ml^−1^ Liberase CI (Roche) and 80 U ml^−1^ DNase I type IV (Sigma-Aldrich). Single-cell suspensions were then prepared by mechanically disrupting the organs through a 70 μm (spleen) or 100 μm (liver) filter. Low-density cells were enriched from the spleen using NycoPrep (1.077 g ml^−1^; Axis-Shield, Oslo, Norway). Liver leukocytes were isolated by centrifugation in 33% Percoll (GE Healthcare, Piscataway, NJ, USA), followed by filtration through a 40 μm cell strainer to remove contaminating *S. mansoni* eggs before RBC lysis. For characterization of phenotypic activation and DC sorting, non-DC lineage cells were then coated with biotinylated monoclonal antibodies against murine CD2, CD3ɛ, CD49b, mIgM and erythrocytes (Ter-119), and depleted using MyOne Streptavidin Dynabeads (Dynabeads Mouse DC Enrichment Kit; Invitrogen). Dead cells were excluded by staining with LIVE/DEAD Fixable Aqua Dead Cell Stain (Invitrogen). After FcR-block (2.4G2), cells were surface stained with combinations of the following monoclonal antibodies: F4/80, NK1.1, CD11c, CD317 (PDCA-1/BST-2), Ly6C, CD11b, MHC class II, CD86 and CD40. Live, non-doublet, F4/80^−^NK1.1^−^ cells that were CD11c^high^CD317^−^ were gated as cDCs, whereas pDCs were defined as CD11c^intermediate^CD317^+^Ly6C^high^CD11b^−^ cells. For CD4^+^ T-cell co-culture experiments, hepatic cDC and pDC populations were sorted from DC-enriched preparations of livers that had been rested overnight at 4 °C, using a BD FACS Aria (San Jose, CA, USA). All antibodies for flow cytometry were purchased from BD Biosciences (San Jose, CA, USA), eBioscience (San Diego, CA, USA), Biolegend (San Diego, CA, USA) or Miltenyi Biotech (Bergisch Gladbach, Germany). Samples were acquired on FACS Canto II or LSR flow cytometers using BD FACS Diva Software and analyzed with FlowJo (Tree Star Inc., Ashland, OR, USA).

### Restimulation assays and intracellular cytokine staining

Single-cell suspensions of liver leukocytes (1 × 10^6^ cells per ml) were cultured in *ex vivo* 15 medium (Lonza, Walkersville, MD, USA) containing 2 mm l-Glutamine and 50 μm 2-ME (Invitrogen) in 96-well plates at 37 °C 5% CO_2_ with or without 15 μg ml^−1^ SEA. After 72 h, supernatants were harvested and analyzed for IL-4, IL-13, IL-10 and IFN-γ using paired capture and detection antibodies (produced from hybridomas in-house or purchased from R&D Systems (Minneapolis, MN, USA), BD Biosciences or eBioscience) and recombinant cytokine standards (Peprotech (Rocky Hill, NJ, USA) or BD Biosciences). For intracellular cytokine staining of liver leukocytes, cells were rested overnight at 4 °C and then stimulated with 10 ng ml^−1^ phorbol myristate acetate and 1 μg ml^−1^ Ionomycin (Sigma-Aldrich) for 2 h, followed by treatment with Golgi stop (BD Biosciences) for an additional 3 h. After FcR-block, cells were surface stained with monoclonal antibodies against CD3 or TCR-β and CD4, fixed with 1% PFA, permeabilized with Perm/Wash buffer (BD Biosciences), and then stained intracellularly with anti-IL-4, anti-IL-13 and anti-IL-10. Identification of cytokine-positive cells was determined using appropriate isotype and Fluorescence Minus One controls (data not shown).

### Ag presentation assays

Naive OT-II CD4^+^ T cells were purified using Dynal Mouse CD4 Cell Negative Isolation Kit (>80% purity; Invitrogen). Pre-activated ‘effector/memory' OT-II CD4^+^ T cells (>90% purity) were generated by culturing OT-II spleen cells in RPMI containing 10% FCS, 200 mm l-Glutamine, 50 μM 2-ME and 1 mg ml^−1^ OVA protein (Sigma-Aldrich) at 37 °C 5% CO_2_ for 7 days. On days 3 and 5, cultures were supplemented with 10 ng ml^−1^ IL-7, 10 ng ml^−1^ IL-15 and 2 ng ml^−1^ IL-2 (Peprotech). Cells were examined by flow cytometry for an effector/memory-like phenotype by surface staining with monoclonal antibodies against CD4, Vα2, CD44 and CD69 (data not shown). For carboxyfluorescein succinimidyl ester (CFSE) labeling, purified OT-II CD4^+^ T cells were labeled with 5 μm CFSE for 15 min at 37 °C, and washed three times before use. For presentation of OVA_323-339_ (OT-II) peptide, FACS-sorted liver DC populations were incubated for 45 min with various concentrations of OVA_323–339_, washed and then 5000 cDCs or pDCs were co-cultured with 50 000 naive or pre-activated CFSE-labeled OT-II cells in 96-well V-bottom plates. For presentation of soluble OVA protein, 5000 cDCs or pDCs were co-cultured with 50 000 naive or pre-activated CFSE-labeled OT-II cells in the presence of various concentrations of endotoxin-depleted soluble OVA protein in 96-well V-bottom plates. After culture for 60–65 h at 37 °C 5% CO_2_, supernatants were harvested for analysis of cytokine production by enzyme-linked immunosorbent assay and OT-II cells were surface stained with anti-CD4 and anti-Vα2 for analysis of proliferation by flow cytometry.

### Statistical analysis

Statistical analysis was performed using a two-tailed Student's *t*-test or one-way analysis of variance with Bonferroni *post hoc* test in Prism (GraphPad Software, Inc., La Jolla, CA, USA). For the *ex vivo* Ag presentation assays, analysis of pooled data from experimental repeats conducted on different days was carried out using a mixed model analysis, labeling the day of the experiment as a random factor (JMP statistical analysis software 11.1.1; SAS Institute Inc., Cary, NC, USA). Differences between groups were determined by analysis of variance followed by a Tukey–Kramer honest significant difference multiple comparison test. Asterisks denote statistically significant differences (*P<0.05; ^**^P<0.01; ^***^P<0.001).
