# Supplementary material for: A central role for hepatic conventional dendritic cells in supporting Th2 responses during helminth infection
Source: Immunol Cell Biol. 2016 Jan 12;94(4):400–10. doi: 10.1038/icb.2015.114 (PMC4817239; doi:10.1038/icb.2015.114)
Supplement: Supplementary Figures [file icb2015114x1.pdf]

Supplementary Figure 1

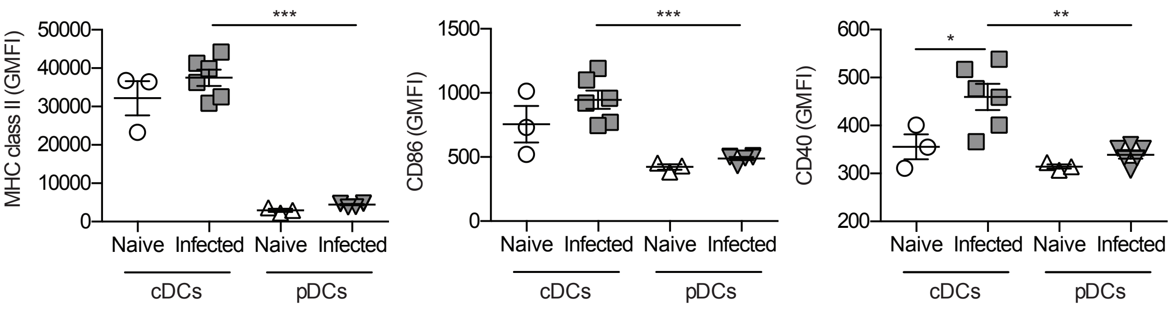

Supplementary Figure 1. Activation phenotype of DCs isolated from the spleen during *S. mansoni* infection. GMFI values for MHC class II, CD86 and CD40 on cDCs and pDCs enriched from the spleens of *S. mansoni*-infected mice (six weeks post-infection). Data are pooled from two experiments. Error bars represent mean ± SEM.

Supplementary Figure 2

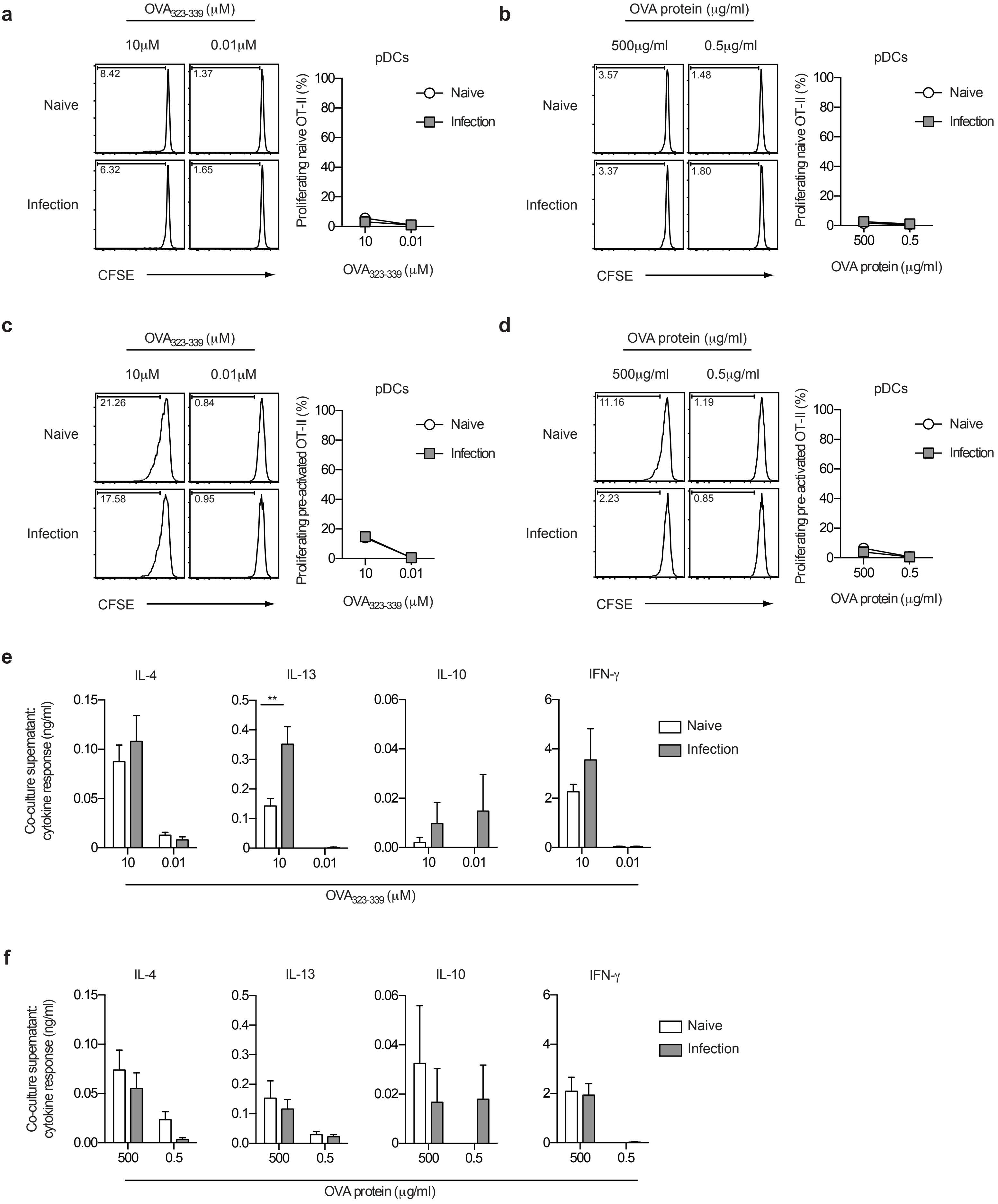

Supplementary Figure 2. Hepatic pDCs are unable to support the expansion of naive and effector/memory $CD4^+$ T cell responses. Proliferation of naïve OT-II $CD4^+$ T cells (A and B) or effector/memory OT-II $CD4^+$ T cells (C and D) in response to presentation of $OVA_{323-339}$ peptide or soluble OVA protein by pDCs isolated from the livers of naïve or infected mice (six weeks post-infection). Cytokine production in supernatants from co-cultures of pDCs and effector/memory OT-II $CD4^+$ T cells (E and F). Data are pooled from three experiments. Error bars indicate mean ± SEM.

Supplementary Figure 3

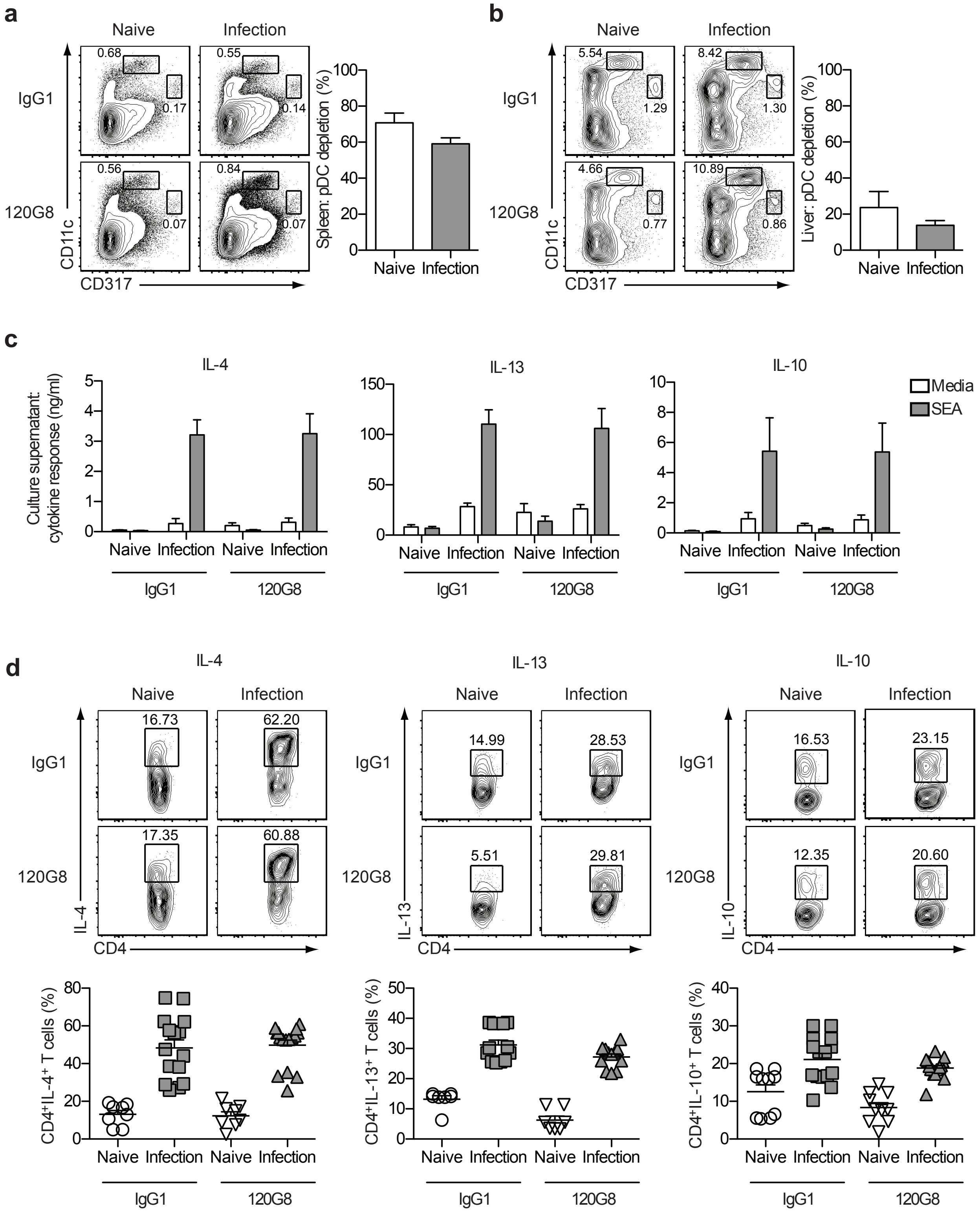

Supplementary Figure 3. Treatment of mice with the pDC-depleting mAb 120G8 has no impact on Th2 responses in the liver. The mAb 120G8 effectively depleted the $CD11c^{int}CD317^{+}$ pDC population in the spleen (A), but was less effective at depleting pDCs in the liver (B), when administered every 48 hours from day 28 to day 42 post-infection. (C) Cells isolated from the livers of naïve or infected IgG1- or 120G8-treated mice were restimulated with medium alone or SEA and supernatants analyzed by ELISA for schistosome egg-specific recall responses. (D) Intracellular cytokine staining was used to directly assess liver $CD4^{+}$ T cell cytokine production. Data are pooled from two to six experiments. Error bars indicate mean ± SEM.

Supplementary Figure 4

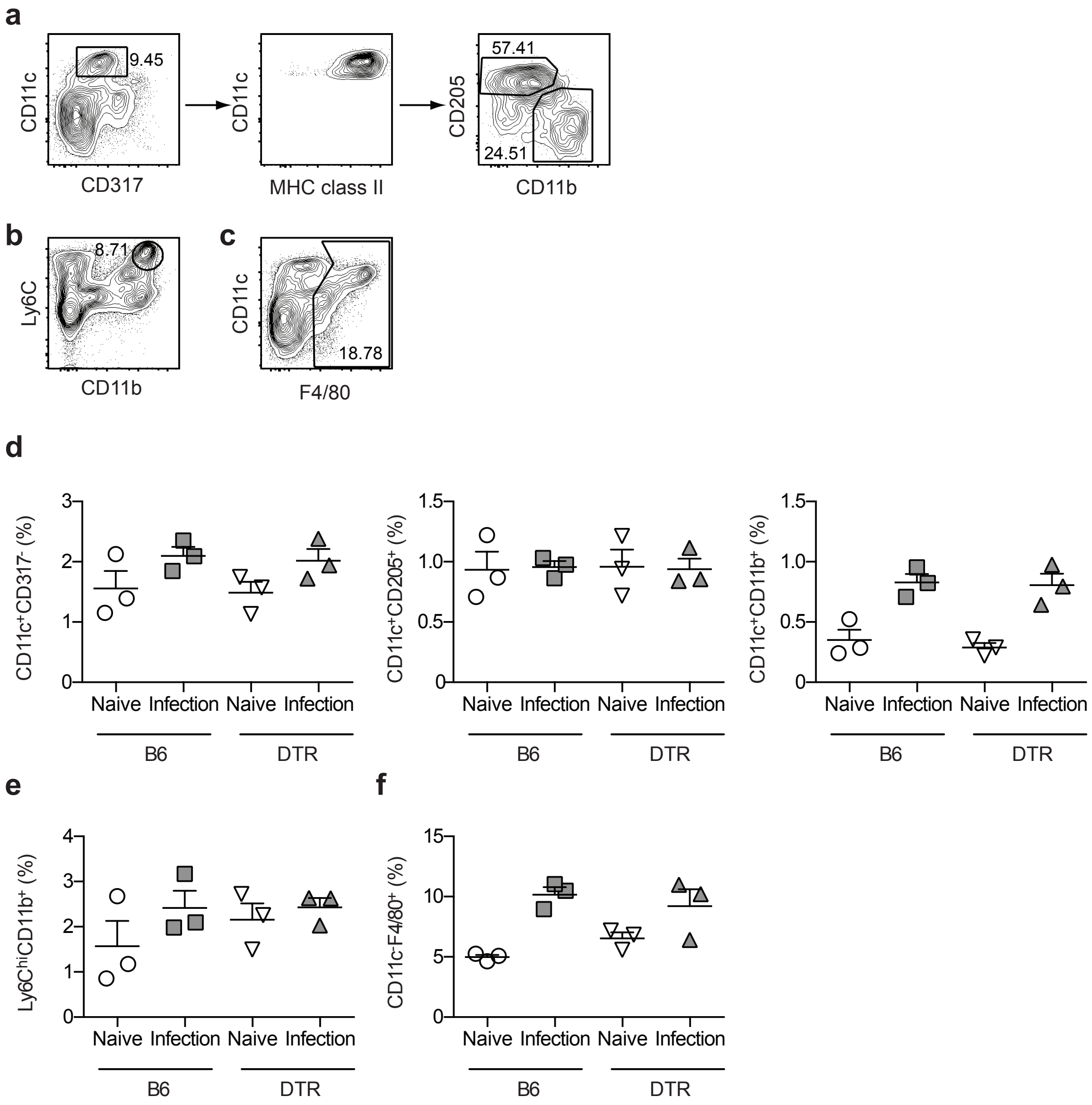

Supplementary Figure 4. Treatment of BDCA2-DTR mice with DT has no impact on non-pDC populations. Gating strategy to identify cDCs (a), Ly6C^{hi} monocytes (b) and F4/80^{+} macrophages (c) in the livers of BDCA2-DTR mice. (d-f) The percentage of each cell population in the livers of naïve or infected DT-treated B6 or BDCA2-DTR mice. Data represent one of three experiments. Error bars indicate mean ± SEM.
